# Designing Antioxidant-Enriched Extracts from *Erica carnea* L.: Optimization, Kinetics, and Thermodynamic Insights

**DOI:** 10.3390/molecules31020245

**Published:** 2026-01-11

**Authors:** Violeta Jevtovic, Khulood Fahad Saud Alabbosh, Buthainah Ameen Al Shankiti, Tarfah Abdulrahman M. Alaskar, Reem Ali Alyami, Walaa I. El-Sofany, Vesna Stankov Jovanović, Jelena Nikolić, Vesna Veličković, Odeh A. O. Alshammari, Milan Mitić

**Affiliations:** 1 Chemistry Department, College of Science, University of Ha’il, Ha’il 81451, Saudi Arabia; v.jevtovic@uoh.edu.sa (V.J.); b.alshanqiti@uoh.edu.sa (B.A.A.S.); t.alaskar@uoh.edu.sa (T.A.M.A.); reem-alyami1@hotmail.com (R.A.A.); wa.ibrahim@uoh.edu.sa (W.I.E.-S.); odeh.alshammari@uoh.edu.sa (O.A.O.A.); 2 Biology Department, College of Science, University of Ha’il, Ha’il 81451, Saudi Arabia; k.alabosh@uoh.edu.sa; 3 Department of Chemistry, Faculty of Science and Mathematics, University of Niš, 18000 Niš, Serbia; vesna.stankov-jovanovic@pmf.edu.rs (V.S.J.); jelena.cvetkovic@pmf.edu.rs (J.N.); 4 Department of Physics and Materials, Faculty of Technical Sciences, University of Kragujevac, 32102 Čačak, Serbia; vesna.velickovic@ftn.kg.ac.rs

**Keywords:** *Erica carnea* L., full factorial design, extraction, antioxidant assays, unsteady-state diffusion model, IC_50_ values, thermodynamic parameters

## Abstract

*Erica carnea* L. has recently gained attention as a promising natural source of antioxidants suitable for food and beverage applications. This study aimed to obtain an antioxidant-enriched extract by optimizing the key extraction variables. A full factorial design was used to evaluate the effects of ethanol concentration, extraction time, and temperature, followed by validation through multiple antioxidant assays, including DPPH, ABTS, hydroxyl radical scavenging, lipid peroxidation inhibition, and metal chelation. The most efficient extraction was achieved at 30% ethanol, 120 min, and 50 °C, yielding IC_50_ values of 18.42 μg/mL (LP), 15.04 μg/mL (DPPH), 5.14 μg/mL (MC), 11.28 μg/mL (OH), and 10.06 μg/mL (ABTS), in agreement with the model predictions. Extraction kinetics were described using an unsteady-state diffusion model, supported by low root mean square (RMS) values and high coefficients of determination. Thermodynamic analysis indicated an irreversible, endothermic process, highlighting the energetic requirements for phenolic release from plant tissue. The combined statistical and kinetic approach provides a clearer understanding of how process variables influence antioxidant recovery. In addition to strong antioxidant activity, the optimized extract showed measurable cytotoxic and antibacterial effects. Overall, these findings highlight *E. carnea* as a valuable material for developing antioxidant-rich formulations, with extraction efficiency governed by predictable kinetic and thermodynamic behavior.

## 1. Introduction

Free radicals are continuously formed in living organisms through endogenous metabolic reactions and by exposure to external factors such as pollutants, xenobiotics, metal ions, and allergens. When their production exceeds the capacity of the cellular defense system, an imbalance known as oxidative stress occurs. Under these conditions, reactive species readily attack proteins, lipids, and nucleic acids, altering their structure and impairing normal cellular functions. Among the various radicals, reactive oxygen species (ROS)—particularly superoxide anions and hydroxyl radicals—are considered the most damaging due to their high reactivity and involvement in pathways leading to inflammation, tissue injury, and programmed cell death [1,2]. Although cells possess endogenous antioxidant mechanisms, excessive ROS accumulation may overwhelm these natural defenses and induce harmful signaling events [3].

An antioxidant is defined as a molecule that, even at low concentrations relative to an oxidizable substrate, can delay or inhibit its oxidation [4]. Interest in natural antioxidants has grown substantially due to their potential to mitigate oxidative stress and their relevance to the prevention of many chronic disorders. Plants represent an abundant source of such compounds, producing a wide array of phenolics and other secondary metabolites with antioxidant and therapeutic properties [5,6,7,8]. Previous studies have reported that *Erica carnea* L., extracts exhibit notable antioxidant, antidiabetic, anti-inflammatory, antibacterial, and analgesic activities [9,10], and were chosen for their high applicability.

The antioxidant response of plant extracts is generally governed by two main mechanisms: hydrogen-atom transfer (HAT) and single electron-transfer (SET). Several commonly applied tests, such as the ABTS and DPPH assays, rely on mixed HAT/SET pathways and are widely used to evaluate the radical-scavenging capacity of natural extracts [11]. Because different assays probe different reaction mechanisms and phases, no single standard method exists for assessing antioxidant activity. Therefore, optimizing extraction conditions for obtaining extracts and applying appropriate analytical antioxidant assays are crucial steps in estimating their antioxidant activity.

Extraction is the first and often most decisive stage in recovering bioactive constituents from plant materials. Solvent composition, extraction temperature, and extraction time all influence the yield of phenolic compounds and ultimately determine the extract’s antioxidant performance. Traditional optimization based on varying one parameter at a time (OVAT) is limited, as it ignores potential interactions among variables and often fails to identify true optimal conditions [12,13]. Statistical approaches, such as full factorial design and response surface methodology, provide a more reliable framework for exploring experimental factors and improving extraction efficiency [14,15]. In parallel, mathematical modeling of extraction kinetics offers insight into mass-transfer behavior and can support the design, control, and scale-up of solid–liquid extraction processes [16,17].

In addition to antioxidant activity, phenolic compounds from plant extracts are often associated with other biologically relevant effects, including antimicrobial and cytotoxic responses. Oxidative stress is closely linked to cellular damage and microbial growth, and compounds capable of scavenging reactive oxygen species may also influence redox-sensitive processes, metal availability, as well as membrane integrity. Since *Erica carnea* L. is known to contain flavonoids and phenolic acids with reported biological effects [9], the inclusion of antibacterial and cytotoxic assessments in this study aimed to provide a broader biological context while maintaining the primary focus on antioxidant optimization.

In the present study, a structured workflow was applied to evaluate and maximize *E. carnea* extracts as a source of bioactive antioxidant compounds. First, extracts were prepared under systematically varied extraction conditions to examine how solvent composition, extraction time, and temperature influence antioxidant activity. A 2^3^ full factorial design was employed to identify the most influential variables and to determine the combination of factors that maximize extract activity. To gain deeper insight into the extraction mechanism, an unsteady-state diffusion model was tested to assess its ability to describe the mass-transfer behavior of antioxidant compounds from the plant matrix. Thermodynamic parameters were also evaluated to clarify the energetic and mechanistic aspects of phenolic release. Finally, extracts obtained under optimal conditions were examined for cytotoxic and antibacterial activity to explore their broader biological potential. This integrated approach provides a comprehensive assessment of *E. carnea* extracts, combining statistical optimization with kinetic and thermodynamic interpretation, and offers a framework for future work on the valorization of these species in food, nutraceutical, and health-related applications.

## 2. Results and Discussion

### 2.1. Antioxidant Activity of E. carnea Extracts

A series of antioxidant assays was used to characterize the activity of the *E. carnea* extracts obtained under different extraction conditions. The five tests applied—lipid peroxidation inhibition, hydroxyl radical scavenging, metal chelation, and the DPPH and ABTS radical scavenging assays—capture different reaction mechanisms and therefore allow a more complete comparison of the extracts’ antioxidant responses. Considerable variation in activity was observed across the extracts, indicating that extraction parameters strongly affect the recovery of antioxidant constituents.

To quantify the contribution of individual factors, the experimental results were evaluated using a 2^3^ full factorial design. The applied design enabled simultaneous analysis of the main extraction variables and their interactions. Each factor was tested at two levels, and data from the initial screening experiments were incorporated into the model. The factorial design thus provided a structured basis for determining which extraction conditions most effectively enhance antioxidant activity. The experimental matrix used in the 2^3^ full factorial design is listed in Table 1, while the experimentally measured and model-predicted responses for all antioxidant assays are presented in Table 2.

Lipid peroxidation is a well-known consequence of oxidative stress and represents one of the major pathways through which ROS compromise membrane integrity and cellular function [18]. In our study, the *E. carnea* extracts displayed notable inhibitory activity in the lipid peroxidation assay, although the extent of protection varied with the extraction conditions. Among the tested samples, the extract prepared with 30% ethanol exhibited the strongest inhibition of lipid peroxidation, indicating that this solvent proportion favored the higher recovery of protective constituents.

Table 2 shows that the DPPH IC_50_ values ranged from 28.94 to 15.09 μg/mL, indicating notable differences in the extracts’ radical-scavenging capacity. Since lower IC_50_ values correspond to stronger antioxidant activity, the most effective DPPH scavenging was observed for the extract obtained using 30% ethanol at 50 °C for 120 min. This superior activity likely reflects a higher recovery of phenolic constituents under these extraction conditions. Similar observations have been reported in previous studies, where strong linear correlations were found between total phenolic content and DPPH radical-scavenging potential [19,20].

The ability of the extracts to chelate ferrous ions (Fe^2+^) was evaluated using the ferrozine assay, which forms a stable-colored complex with free iron. As shown in Table 2, the extracts exhibited noticeable variation in metal-chelating capacity under different extraction conditions. The strongest chelating activity (IC_50_ = 5.14 μg/mL) was observed in the extract prepared with 30% ethanol at 50 °C for 120 min, indicating efficient recovery of compounds capable of binding Fe^2+^. In contrast, the extract produced with 50% ethanol at 30 °C for 40 min exhibited the weakest activity, with an IC_50_ value of 19.87 μg/mL. These suggest that lower ethanol concentration and longer extraction times were more effective for isolating iron-chelating constituents and vice versa.

Due to their very short lifetime in vivo (≈10^−9^ s), hydroxyl radicals (∙OH) readily interact with surrounding biomolecules, contributing to oxidative damage. [21]. It can initiate oxidative modifications of lipids, proteins, and nucleic acids, ultimately contributing to cellular dysfunction. In our study, the ∙OH-scavenging ability of the *E. carnea* extracts, expressed as IC_50_ values, ranged from 20.22 to 11.28 μg/mL. The strongest activity again corresponded to the extract obtained with 30% ethanol at 50 °C for 120 min. This effect may be associated with the greater recovery of flavonoid constituents, particularly quercetin derivatives previously reported in *E. carnea* by Veličković et al. (2017) [9].

To complement the antioxidant evaluation, the ABTS test was applied, as it allows the assessment of antioxidant activity across compounds with differing solubility properties [22]. As shown in Table 2, ABTS IC_50_ values ranged from 10.06 to 19.44 μg/mL. As observed in the DPPH and hydroxyl radical assays, the extract prepared with 30% ethanol at 50 °C for 120 min exhibited the highest activity. Notably, the extract obtained at the same ethanol concentration and extraction time but at a lower temperature (30 °C) showed a very similar value (11.03 μg/mL), indicating a relatively stable performance of these conditions across different assays.

The variability observed among the different antioxidant tests reflects the complex composition of the extracts and the diversity of mechanisms involved in radical neutralization. Each assay is based on a distinct reaction pathway, and different classes of phytochemicals may dominate the activity depending on the radical species assessed. While polyphenols—especially flavonoids—are typically considered the main contributors to antioxidant capacity, additive, synergistic or antagonistic interactions with other secondary metabolites may also influence the overall response.

### 2.2. Pearson Correlations

The Pearson correlation analysis was used to examine the relationships among all dependent variables in the full factorial design. This statistical method quantifies the strength of linear association between pairs of variables, providing insight into whether different antioxidant assays respond similarly to changes in extraction conditions.

The correlation coefficients for the antioxidant activities of *E. carnea* extracts are presented in Table 3. As expected, lipid peroxidation (LP) showed strong positive correlations with both metal-chelating activity (MC) and DPPH radical scavenging, with R^2^ values of 0.9808 and 0.9793, respectively. A similarly strong correlation was observed between the DPPH and ABTS assays (R^2^ = 0.9716). These patterns reflect the shared underlying mechanisms of phenolic antioxidants, which primarily act through hydrogen-atom and electron-transfer pathways.

These findings are consistent with our previous work [10], in which extracts obtained with 30% ethanol at 50 °C (after 80 min of extraction) exhibited similar correlations. Together, these results reinforce the conclusion that the dominant bioactive constituents in *E. carnea* extracts contribute broadly to all measured antioxidant activities, regardless of the assay employed.

Earlier studies on *E. carnea* L. have applied extraction protocols characterized by high ethanol concentrations and extended maceration times, in contrast to the conditions examined here [9]. That study identified several phenolic acids—most notably chlorogenic (0.132 mg/g), vanillic (0.129 mg/g), ferulic (0.113 mg/g), and *p*-coumaric acid (0.108 mg/g)—as well as flavonoids such as rutin (2.159 mg/g), quercetin (1.895 mg/g), luteolin (0.559 mg/g), and apigenin (0.321 mg/g). These compounds are well known for their strong antioxidant potential, which provides further support for the correlations observed in the present study. All pairwise correlations were statistically significant (*p* < 0.001), confirming that all antioxidant assays responded consistently to variations in extraction conditions.

### 2.3. Effect of Extraction Parameters on Antioxidant Activity

The extent to which phenolic compounds are released from plant tissues into the extraction medium is influenced by their affinity for the solvent, with solvent polarity playing a key role in this process. Selecting an appropriate solvent system directly affects mass transfer from the solid matrix to the liquid phase [23]. Based on preliminary tests, ethanol–water mixtures containing 30% and 50% ethanol were selected for further experiments.

For potential food or pharmaceutical use, water and ethanol represent suitable extraction solvents due to their non-toxicity, food-grade status, low cost, and environmental compatibility [24]. Accordingly, different ethanol–water ratios were evaluated to determine the most effective solvent composition for recovering compounds with antioxidant activity.

To identify which extraction parameters most strongly influenced antioxidant responses, an analysis of variance (ANOVA) was performed (Table 4). The statistical significance of the main effects and their combined effects were evaluated using F-values and *p*-values, where larger F-values indicate stronger contributions to the model, and *p* < 0.05 denotes statistical significance. Among all terms, the linear effect of ethanol concentration (x_1_) was the most influential (F = 197.62–2764.4, *p* < 0.00001). Extraction time (x_3_) was the next most significant factor (F = 96.449–1393.1, *p* < 0.00001), followed by extraction temperature (x_2_), which also contributed meaningfully to the model (F = 23.122–518.22, *p* < 0.00001 or 0.002, depending on the response).

**Figure 1:**
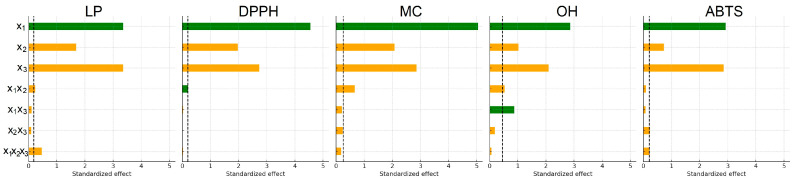
Pareto charts of factors influencing the antioxidant activity of extracts: x_1_, ethanol concentration; x_2_, extraction temperature; x_3_, extraction time. (Green bars indicate positive standardized effects, while orange bars represent negative effects. All effects are shown as absolute values, and the dashed line indicates the significance limit for standardized effects).

To visualize the relative contribution of individual factors, Pareto charts of standardized effects were generated for all antioxidant assays (Figure 1). These charts identify which factors exceed the 95% confidence threshold and therefore contribute significantly to the model.

**Figure 2:**
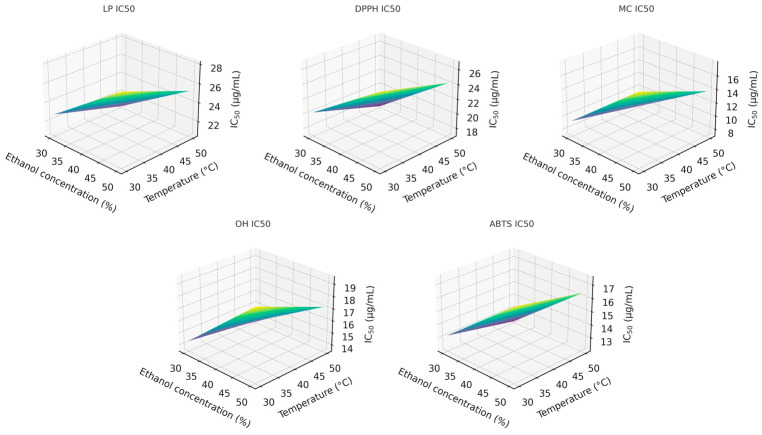
Response-surface plots for antioxidant compounds extraction with: 1-LP; 2-DPPH; 3-MC; 4-OH, and 5-ABTS activity.

To better understand the interaction between ethanol concentration and extraction temperature, three-dimensional response-surface plots were produced at the midpoint extraction time (80 min). These surfaces clearly illustrate the joint influence of the two factors on the IC_50_ values across all antioxidant assays (Figure 2).

Among the factors analyzed, ethanol concentration (x_1_) exerted the strongest influence on the extraction of compounds responsible for the DPPH, MC, and OH activities, followed by extraction time (x_3_). For LP and ABTS, the trend differed slightly, following the order x_1_ ≈ x_3_ > x_2_. The two-way interaction between ethanol concentration and temperature (x_1_x_2_) significantly affected the LP, DPPH, MC, and OH responses but showed no meaningful effect on ABTS activity. No statistically significant combined effect of all three factors was observed for DPPH, MC, or OH responses. However, it was statistically significant (*p* < 0.05) for LP and ABTS, indicating more complex factor interdependence in these two antioxidant assays.

Regression equations were developed using coded factors, incorporating both linear and interaction terms to describe the relationships between extraction parameters and antioxidant responses. These models provide a detailed understanding of how processing conditions influence LP, DPPH, MC, OH, and ABTS activities and offer predictive capability useful for optimizing extraction conditions in future applications.

The final regression equations expressed in terms of coded variables are summarized in Table 5. The predicted response values from these equations (Table 5) were compared with the experimental data in Table 1. The consistency between experimentally obtained data and model predictions indicates that the polynomial models reliably capture the antioxidant responses within the investigated extraction range.

An R^2^ value above 97% further confirms the excellent fit of the developed models. The adjusted R^2^ values exceeded 95% for all responses, while the coefficients of variation (CVs) were below 3.2%, collectively demonstrating the high reliability, precision, and overall goodness of fit of the regression models.

### 2.4. Extraction Kinetics of Antioxidants

#### Antioxidant Activity Variation with Extraction Time

**Figure 3:**
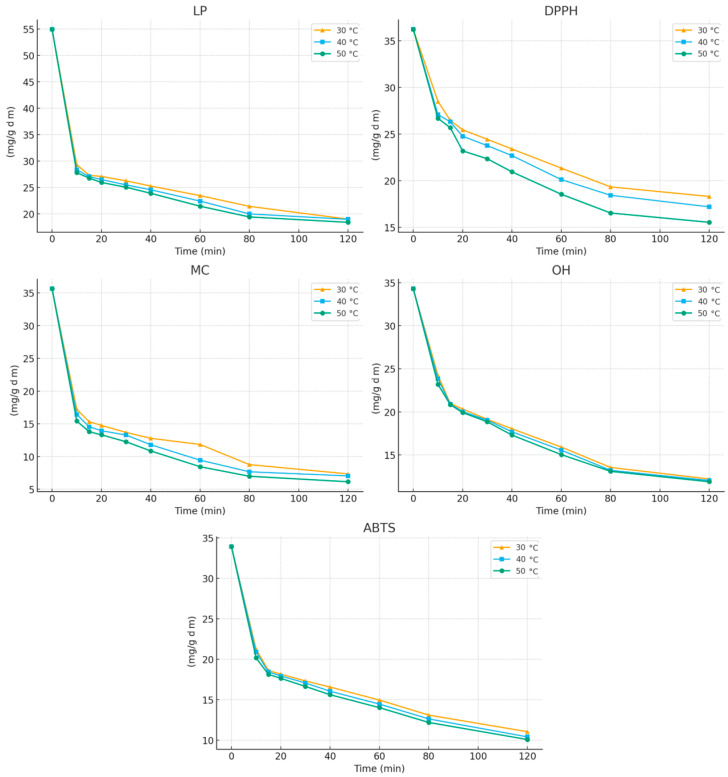
Variation in antioxidant activity of extracts during the extraction process at (▲) 30 °C; (▪) 40 °C; and (•) 50 °C.

Following this analysis, the kinetic and thermodynamic parameters governing the extraction of antioxidant compounds from *E. carnea* were evaluated. Figure 3 presents the time-dependent changes in antioxidant activity during extraction with 30% ethanol, monitored using the different antioxidant assays at various temperatures.

An increase in extraction time from 10 to 120 min resulted in a progressive enhancement of all antioxidant activities, with the highest activity consistently observed at 120 min. The extraction curves reveal two distinct phases characteristic of solid–liquid extraction processes:

(a) An initial rapid release of c (the washing phase), where easily accessible constituents quickly diffuse into the solvent, and
(b) A subsequent slower phase (the diffusion-controlled phase), occurring after approximately 15 min, during which the transfer of compounds is governed by internal mass-transfer resistance within the plant matrix.

During the first 15 min of extraction, a pronounced decrease in the initial IC_50_ values was observed, corresponding to increases in antioxidant activity of approximately 50%, 27%, 57%, 39%, and 45% for LP, DPPH, MC, OH, and ABTS assays, respectively (at 30 °C). After this rapid initial stage, the extraction rate slowed markedly as internal diffusion became the dominant mechanism. This biphasic behavior—an initial “washing” phase followed by a slower diffusion-controlled phase—has similarly been reported for the extraction of polyphenols from grape seeds [25].

To quantitatively characterize the extraction process, the experimental kinetic data were modeled using an unsteady-state diffusion approach. kinetic parameters were obtained by linear regression of the linearized model equation, and the resulting values are summarized in Table 6. Both kinetic constants b and k increased with temperature, consistent with the experimentally observed enhancement of antioxidant activity at higher temperatures.

Comparison of washing and diffusion rates from different antioxidant assays showed that each assay yielded distinct rate constants. This variation reflects the fact that each assay probes a different chemical mechanism and is therefore sensitive to different groups of antioxidant compounds.

The solvent composition exerted a strong effect on kinetic behavior. When 50% ethanol was used instead of 30% ethanol, both b and k decreased across all assays. On average, the washing-rate parameter decreased by 29.7%, 57.3%, 19.0%, 9.0%, and 12.5% for LP, DPPH, MC, OH, and ABTS, respectively. The diffusion-rate constant *k* decreased by 5.8%, 13.9%, 25.6%, 10.7%, and 19.7% for the same assays. These reductions are fully consistent with the extraction optimization results, further confirming that 30% ethanol is more effective than 50% ethanol for recovering *E. carnea* antioxidant constituents.

Model adequacy was assessed using the coefficient of determination (R^2^) and root mean square error (RMS). As summarized in Table 6, R^2^ values were higher than 0.80 for all responses, while RMS values were below ±10%, indicating that the unsteady-state diffusion model provides a satisfactory description of the extraction kinetics.

### 2.5. Thermodynamics Study of Extracted E. carnea Antioxidants

Activation energy (Ea) is the minimum energy required for an extraction process to proceed [26]. It is a key parameter for understanding the ease or difficulty with which bioactive compounds are released from the plant matrix [27]. The magnitude of Ea may be affected by multiple factors, such as structural characteristics of the plant material, the chemical nature of the target compounds, sample pretreatment, and the type of solvent employed [28].

The effect of temperature on the washing and diffusion-controlled stages of antioxidant extraction was evaluated using the Arrhenius equation (Equation (4)). Its linearized form (Equation (5)) allowed the relationship between temperature (T) and the kinetic constants b and k to be determined. The calculated activation energies and related thermodynamic parameters for the solid–liquid extraction of *E. carnea* are presented in Table 7.

The activation energies obtained were positive for all assays, confirming that the extraction of antioxidants from *E. carnea* is an endothermic process. In agreement with the kinetic analysis, the rate constants increased with rising temperature, indicating that temperature favors both the initial and the diffusion-controlled stages of extraction.

For the washing phase, the calculated activation energies were 3.60 kJ·mol^−1^ for LP, 3.98 kJ·mol^−1^ for DPPH, 2.29 kJ·mol^−1^ for MC, 2.38 kJ·mol^−1^ for OH, and 2.53 kJ·mol^−1^ for ABTS. These values show that the initial extraction rate was most sensitive to temperature for compounds exhibiting DPPH activity, whereas the lowest sensitivity occurred for those contributing to MC activity.

For the slow, diffusion-controlled stage, the activation energies were higher: 7.51 kJ·mol^−1^ for LP, 4.97 kJ·mol^−1^ for DPPH, 3.22 kJ·mol^−1^ for MC, 3.63 kJ·mol^−1^ for OH, and 5.67 kJ·mol^−1^ for ABTS. In contrast to the washing phase, the slow extraction rate showed the greatest temperature sensitivity for LP, while MC again exhibited the lowest sensitivity. These results reinforce the observation that different antioxidant assays reflect the extraction behavior of different classes of compounds, each responding uniquely to temperature.

**Figure 4:**
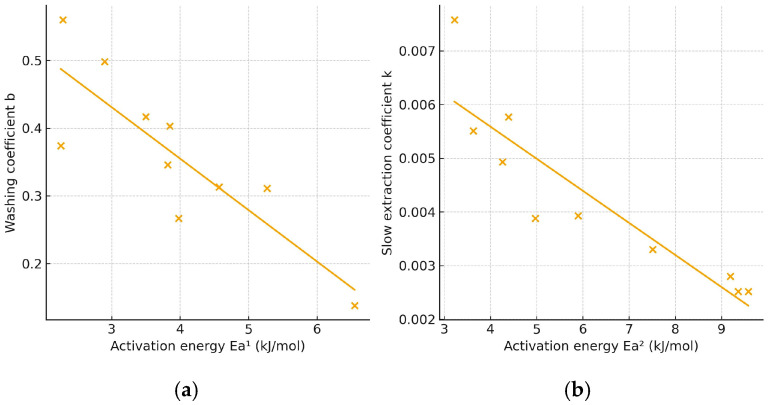
Plot of activation energy vs. washing (**a**) and slow extraction coefficient (**b**).

Overall, lower activation energy corresponds to a faster extraction rate, as a greater proportion of molecules can effectively contribute to mass transfer under the applied conditions. Figure 4 illustrates the relationship between the mean values of the washing coefficient (b) or the slow-extraction coefficient (k) and the corresponding activation energies for extraction with 30% and 50% ethanol. The correlation analysis revealed a significant (*p* < 0.05) negative linear relationship between activation energy and both kinetic parameters: *r* = −0.9111 for Ea vs. b and *r* = −0.9017 for Ea vs. k. The negative slopes indicate that an increase in activation energy is associated with a decrease in the values of b and k, confirming that higher energy barriers slow both the initial and diffusion-controlled stages of the extraction process.

The activation energies calculated for the extraction process using 30% ethanol were consistently lower than those obtained with 50% ethanol, irrespective of the antioxidant assay applied. This finding further supports that 30% ethanol is a more efficient solvent for extracting antioxidants from *E. carnea*, as it promotes faster extraction and higher recovery of bioactive compounds.

Activation enthalpy, entropy, and Gibbs free energy were estimated at each temperature according to Equations (4)–(6). The positive ΔH* values confirmed that the extraction of total polyphenols and antioxidant-active compounds is an endothermic process, requiring external energy input to transition molecules into the activated state. In contrast, all ΔS* values were negative under the experimental conditions. Such negative entropy values suggest a transition state of reduced molecular disorder, typically associated with the formation of an activated complex in which reactant species lose degrees of freedom during the extraction process [16,29]. A more negative ΔS* therefore indicates a more ordered transition state and a slower extraction rate.

The spontaneity of the extraction process was evaluated using the Gibbs free energy of activation (ΔG*). The positive ΔG* values obtained at all temperatures demonstrated that the extraction of antioxidants from *E. carnea* is an endergonic and non-spontaneous process, requiring thermal energy to proceed.

### 2.6. Biological Activity of Obtained Extract

The extracts prepared under different extraction conditions—ethanol concentrations of 30% and 50%, temperatures of 30 °C, 40 °C, and 50 °C, and an extraction time of 120 min—were subsequently evaluated for their cytotoxic and antibacterial activities.

#### 2.6.1. Cytotoxic Activity

The cytotoxic effects of the obtained extracts on the three investigated cell lines are presented in Table 8. Overall, extracts prepared with 30% ethanol showed the highest cytotoxic potential across all cell lines. In addition, increasing the extraction temperature consistently enhanced the cytotoxic effect, independent of the ethanol concentration used.

#### 2.6.2. Antimicrobial Activity

Antimicrobial effects of the prepared extracts were assessed against selected bacterial and fungal strains, with the results summarized in Table 9. The tested microorganisms displayed varying degrees of sensitivity to the extracts. In general, extracts obtained using 30% ethanol demonstrated the strongest antibacterial effects across most strains.

### 2.7. Phenolic Profile of the Optimized Extract

To complement the extraction optimization, the chemical composition of the extract obtained under optimal conditions (30% ethanol, 120 min, 50 °C) was analyzed using HPLC-DAD. The analysis confirmed the presence of several dominant phenolic constituents, including rutin, quercetin, luteolin, apigenin, rosmarinic acid, and sinapic acid. Additional phenolic acids detected were *p*-hydroxybenzoic, vanillic, chlorogenic, and *p*-coumaric acids. Their relative abundance followed the order: rutin (2.315 mg/g) > quercetin (1.935 mg/g) > rosmarinic acid (0.937 mg/g) > luteolin (0.662 mg/g) > sinapic acid (0.442 mg/g) > apigenin (0.352 mg/g), indicating that rutin is the major contributor to the phenolic profile of the extract.

The extraction of *Erica carnea* L. has been previously examined by Veličković et al. [9] using substantially different conditions, including high ethanol concentration, ambient temperature, and prolonged maceration. That study identified several phenolic acids, with rosmarinic acid (0.746 mg/g) and sinapic acid (0.408 mg/g) as the most abundant, along with chlorogenic and *p*-coumaric acids. Among flavonoids, rutin (2.159 mg/g), quercetin (1.895 mg/g), luteolin (0.559 mg/g), and apigenin (0.321 mg/g) were predominant. The lower phenolic content reported in that study compared to our results is likely due to the reduced solubility of phenolics in 96% ethanol and the prolonged maceration period, during which partial degradation of sensitive compounds may occur.

## 3. Materials and Methods

### 3.1. Chemicals and Reagents

All chemicals and reagents employed in this work were of analytical grade. They were purchased from Sigma (Sigma-Aldrich GmbH, Steinheim, Germany) and Sigma (St. Louis, MO, USA).

### 3.2. Plant Material

Aerial parts of spring heath (*Erica carnea* L.) were collected in the Čačak region, Republic of Serbia. Plant identification was performed by a qualified botanist, and a voucher specimen was deposited at the Institute of Biology, Faculty of Science, University of Kragujevac (No. 126/017). The collected material was naturally dried in the shade under well-ventilated conditions at ambient temperature for one month. After drying, the plant material was ground to a uniform particle size using a laboratory blender and stored in paper bags in a dry and dark place until further analysis.

### 3.3. Preparation of Extracts

Extracts were prepared by maceration. Plant material (2.5 g) was extracted with ethanol–water mixtures containing 30% or 50% ethanol, using a fixed solid-to-solvent ratio of 1:20 (g/mL). The maceration was performed in sealed glass vessels placed in a thermostated water bath, with occasional manual agitation. The extraction was carried out at controlled temperatures of 30 °C, 40 °C, or 50 °C for 120 min, with occasional shaking to facilitate mass transfer. After extraction, the mixtures were filtered through Whatman No. 1 filter paper and evaporated to dryness using a rotary evaporator (Devarot, Elektromedicina, Ljubljana, Slovenia). The obtained dry extracts were stored in dark glass bottles at 4 °C to prevent oxidative degradation.

### 3.4. Experimental Design

Optimization of antioxidant activity was performed using a 2^3^ full factorial experimental design (Table 1). The independent variables were solvent composition (30% and 50% ethanol in water), extraction temperature (30 and 50 °C), and extraction time (40 and 120 min). The responses evaluated were the antioxidant activities of the extracts, determined using LP, DPPH, MC, OH, and ABTS assays.

The experimental data were fitted to a first-order linear polynomial model (Equation (1)):

(1) $$y=b_{o}+b_{1}x_{1}+b_{2}x_{2}+b_{3}x_{3}+b_{12}x_{1}x_{2}+b_{13}x_{1}x_{3}+b_{23}x_{2}x_{3}+b_{123}x_{1}x_{2}x_{3}$$

Here, $x_{1}$, $x_{2}$, and $x_{3}$ correspond to ethanol concentration, extraction temperature, and extraction time, respectively. The interaction terms $x_{1}x_{2}$, $x_{1}x_{3}$, and $x_{2}x_{3}$ represent two-factor interactions, whereas the term $x_{1}x_{2}x_{3}$ represents the combined interaction of all three variables. In the model, $b_{0}$ is the intercept, $b_{i}$ is the linear coefficient, and $b_{ij}$ and $b_{ijk}$ are the coefficients describing two- and three-factor interactions.

Regression coefficients were obtained using a multiple linear modeling approach. The statistical significance of each model term was assessed by analysis of variance (ANOVA) at a 95% confidence level (*p* < 0.05). Terms with *p* < 0.05 were considered statistically significant.

Model adequacy and predictive quality were evaluated using the coefficient of determination (R^2^), adjusted R^2^ (R^2^_adj_), and the coefficient of variation (CV).

### 3.5. Determination of Antioxidant Activity

A range of standard in vitro tests was employed to examine the antioxidant potential of the extracts, covering lipid peroxidation inhibition, hydroxyl radical scavenging, DPPH and ABTS radical scavenging, as well as metal-ion chelating activity [30,31,32,33,34]. The results were expressed as IC_50_ values (μg/mL).

### 3.6. Determination of Biological Activity of Extracts

The biological activity of the obtained extracts was evaluated using established in vitro methods. Cytotoxic activity was assessed on human cancer cell lines (HeLa, A549 and LS174T) and normal human lung fibroblasts (MRC-5) using the MTT assay, following the procedure described by Radojković et al. [35]. Cell viability was determined after exposure to different extract concentrations, and the results were expressed as IC_50_ values.

Antimicrobial activity was evaluated by the microdilution method in 96-well microtiter plates, according to previously published protocols [36,37]. The antibacterial activity was tested against selected Gram-positive and Gram-negative bacterial strains, while antifungal activity was assessed against *Candida albicans* and *Aspergillus niger*. Minimum inhibitory concentrations (MICs) were determined as the lowest extract concentration inhibiting visible microbial growth. Reference antimicrobial agents were used as positive controls. Tetracycline hydrochloride (commercial name: Amracin) and nystatin were used as positive controls for antibacterial and antifungal assays, respectively.

### 3.7. Kinetics of Extraction

Kinetic experiments were performed by placing plant material (2.5 g) and 50 mL of aqueous ethanol (30% *v*/*v*) into a series of 250 mL Erlenmeyer flasks. The flasks were maintained at a controlled temperature of 30 ± 0.1 °C, and extraction was carried out for predefined time intervals (10, 15, 20, 30, 40, 60, 80, and 120 min). At the end of each interval, the liquid extract was separated from the plant material by vacuum filtration, after which its antioxidant activity was determined. Each extraction point was prepared in triplicate, and the corresponding values were averaged.

The procedure was repeated using 50% ethanol as the extraction solvent at temperatures of 40 ± 0.1 °C and 50 ± 0.1 °C to evaluate the influence of solvent composition and temperature on extraction kinetics.

### 3.8. Modeling of Antioxidant Extraction Kinetics

The extraction kinetics of compounds exhibiting antioxidant activity was evaluated using the unsteady-state diffusion model. These kinetic parameters provide important insight into the extraction efficiency and mass-transfer characteristics of bioactive constituents from *Erica carnea* L.

The unsteady-state diffusion model is expressed in Equation (2) and can be rearranged as a linear Equation (3) [38]:

(2) $${AA}_{t}/{AA}_{o}=\left(1-b\right)e^{-kt}$$

(3) $$ln\left({AA}_{t}/{AA}_{o}\right)=ln\left(1-b\right)-kt$$

In this expression, AAₜ denotes the antioxidant activity of the liquid extract at time t (IC_50_, μg/mL), while AA_0_ represents the initial antioxidant activity. The parameter k corresponds to the slow extraction rate constant of the unsteady-state diffusion model (min^−1^), whereas b describes the washing coefficient of the same model.

### 3.9. Determination of Thermodynamic Parameters

The temperature dependence of the slow extraction coefficient (k) was analyzed according to the Arrhenius approach, allowing the estimation of activation energy. The nonlinear and linear forms of this relationship are presented in Equations (4) and (5) [16]:

(4) $$k=Ae^{-E_{a}/RT}$$

(5) $$lnk=\frac{-E_{a}}{R}\left(\frac{1}{T}\right)+lnA$$

In these expressions, Ea denotes the activation energy (kJ/mol), R is the universal gas constant, and T represents the absolute temperature (K).

The relationship between ln k and 1/T was used to extract kinetic parameters, where the slope corresponds to −Ea/R and the intercept reflects the Arrhenius constant (ln A). Thermodynamic activation parameters were subsequently determined using the following equations:

(6) $$∆H^{*}=E_{a}-RT$$

(7) $$∆G^{*}=∆H^{*}-T∆S^{*}$$

In this context, ΔH* represents the activation enthalpy, ΔS* the activation entropy, and ΔG* the Gibbs free energy of activation.

### 3.10. HPLC Analysis

The phenolic profile of the optimized extract was determined by a reversed-phase HPLC-DAD method following Veličković et al. [9]. Briefly, 5 μL of sample was injected into an Agilent 1200 system (Agilent Technologies, Santa Clara, CA, USA) equipped with a DAD detector and separated on an Eclipse XDB-C18 column (150 × 4.6 mm, 5 μm) at 30 °C, with a flow rate of 0.8 mL/min. Gradient elution was performed using solvent A (water + 5% formic acid) and solvent B (acetonitrile/water/formic acid, 80:15:5). Phenolic compounds were identified by comparing retention times and UV spectra with standards, and quantified using external calibration.

### 3.11. Statistical Analysis

All experiments were performed in triplicate, and the results are presented as mean ± standard deviation. Statistical significance was evaluated using analysis of variance (ANOVA) at a 95% confidence level (*p* < 0.05). Regression analysis and model fitting were performed using MATLAB (R2022b, MathWorks, Natick, MA, USA).

## 4. Conclusions

This study demonstrated that extraction conditions strongly determine the antioxidant, cytotoxic, and antibacterial activities of *Erica carnea* extracts. Among the tested parameters, ethanol concentration and extraction time had the greatest influence, with 30% ethanol consistently providing higher antioxidant activity and faster extraction rates than 50% ethanol. Kinetic modeling confirmed a two-stage mechanism—a rapid washing phase followed by slower diffusion, while thermodynamic analysis showed that the extraction is endothermic and temperature-dependent. Extracts obtained under optimized conditions also exhibited the strongest biological activity, indicating their potential use in functional food, nutraceutical, and pharmaceutical applications. The predictive models developed here offer a reliable basis for optimizing future extraction processes and support the broader valorization of *E. carnea* as a natural source of bioactive compounds.

From a practical standpoint, these findings provide a useful framework for developing antioxidant-rich extracts using simple and cost-effective extraction conditions. The optimized maceration parameters identified here are well suited for scale-up and could be readily applied in industrial settings. Furthermore, the combined kinetic and thermodynamic approach may be extended to other plant materials, offering a rational basis for future extraction optimization and supporting further research on formulation performance and biological efficacy.

## Figures and Tables

**Table 1:** Full factorial 2^3^ experimental design showing coded and actual values of the independent variables used for extraction.

No	Independent Variables		
	x_1_ (%)	x_2_ (°C)	x_3_ (min)
1	30 (−1)	30 (−1)	40 (−1)
2	50 (+1)	30 (−1)	40 (−1)
3	30 (−1)	50 (+1)	40 (−1)
4	50 (+1)	50 (+1)	40 (−1)
5	30 (−1)	30 (−1)	120 (+1)
6	50 (+1)	30 (−1)	120 (+1)
7	30 (−1)	50 (+1)	120 (+1)
8	50 (+1)	50 (+1)	120 (+1)

**Table 2:** Experimentally measured and model-predicted antioxidant responses (IC_50_ values) obtained under different extraction conditions.

No	Measured Responses									
	LP ^1^_ex_	LP_pred_	DPPH ^2^_ex_	DPPH_pred_	MC ^3^_ex_	MC_pred_	OH ^4^_ex_	OH_pred_	ABTS ^5^_ex_	ABTS_pred_
1	25.48 ± 0.40	25.62	22.41 ± 0.90	22.11	11.23 ± 0.84	11.43	16.46 ± 0.20	16.54	15.17 ± 0.33	15.30
2	30.32 ± 0.28	30.31	28.94 ± 0.17	28.94	19.87 ± 0.33	20.02	20.22 ± 0.62	20.40	19.44 ± 0.25	19.31
3	22.73 ± 0.35	22.74	19.12 ± 0.89	19.16	9.20 ± 0.33	9.32	15.90 ± 0.43	15.81	14.24 ± 0.16	14.22
4	28.27 ± 0.89	28.13	26.33 ± 0.12	26.29	16.37 ± 0.67	15.89	18.26 ± 0.29	18.08	18.88 ± 0.23	18.89
5	20.03 ± 0.36	19.90	18.32 ± 0.60	18.31	7.34 ± 0.10	7.15	12.20 ± 0.75	12.11	11.03 ± 0.28	11.05
6	25.96 ± 0.22	25.97	24.83 ± 0.70	24.83	15.80 ± 0.44	15.73	18.76 ± 0.13	18.58	15.73 ± 0.56	15.71
7	18.42 ± 0.37	18.40	15.09 ± 0.90	15.05	5.14 ± 0.17	5.03	11.28 ± 0.14	11.36	10.06 ± 0.15	9.93
8	22.24 ± 0.17	22.38	22.14 ± 0.79	22.18	11.13 ± 0.29	11.59	16.06 ± 0.75	16.24	13.80 ± 0.37	13.93

^1^ Lipid peroxidation assay (IC_50_, μg/mL); ^2^ DPPH radical scavenging activity (IC_50_, μg/mL); ^3^ Metal chelating activity (IC_50_, μg/mL); ^4^ Hydroxyl radical scavenging activity (IC_50_, μg/mL); ^5^ ABTS scavenging activity (IC_50_, μg/mL).

**Table 3:** Pearson correlation analysis between different antioxidant activity assays.

Test	LP.	DPPH	MC	OH	ABTS
LP	1				
DPPH	0.9793	1			
MC	0.9808	0.9658	1		
OH	0.9425	0.8867	0.9286	1	
ABTS	0.9393	0.9716	0.9518	0.8508	1

LP, lipid peroxidation inhibition; DPPH, DPPH radical scavenging activity; MC, metal chelating activity; OH, hydroxyl radical scavenging activity; ABTS, ABTS radical scavenging activity.

**Table 4:** Analysis of variance (ANOVA) results for the fitted first-order polynomial models.

	SOV	*x*_1_	*x*_2_	*x*_3_	*x*_1_ *x*_2_	*x*_1_ *x*_3_	*x*_2_ *x*_3_	*x*_1_ *x*_2_ *x*_3_	Error	Total
LP	SS ^1^	50.652	12.827	50.752	0.2485	0.0496	0.0351	0.9870		
	Df ^2^	1	1	1	1	1	1	1	0.2915	115.84
	MS ^3^	50.652	12.872	50.752	0.2485	0.0496	0.0351	0.9870	8	15
	F	1390.4	352.10	1393.1	6.8213	1.3617	0.9637	27.093	0.03643	7.7229
	*p*	<0.00001 ^4^	<0.00001	<0.00001	0.031045	0.276847	0.355009	0.000817		
DPPH	SS	93.161	17.464	33.702	0.1860	0.0041	0.0001	0.0025		
	Df	1	1	1	1	1	1	1	0.2696	144.79
	MS	93.161	17.464	33.702	0.1860	0.0041	0.0001	0.0025	8	15
	F	2764.4	518.22	1000.1	5.5207	0.1202	0.0014	0.0727	0.0337	9.6526
	*p*	<0.00001	<0.00001	<0.00001	0.046709	0.737865	0.97107	0.79427		
MC	SS	114.68	19.500	36.851	2.0301	0.2016	0.2556	0.1485		
	Df	1	1	1	1	1	1	1	1.1776	148.85
	MS	114.68	19.500	36.851	2.0301	0.2016	0.2556	0.1485	8	15
	F	779.11	132.47	250.35	13.798	1.3696	1.7364	1.0088	0.1472	11.657
	*p*	<0.00001	<0.00001	<0.00001	0.005917	0.275565	0.224078	0.344599		
OH	SS	38.237	4.7124	19.656	1.2640	3.4060	0.1512	0.0018		
	Df	1	1	1	1	1	1	1	1.6304	69.0597
	MS	38.237	4.7124	19.656	1.2640	3.4060	0.1512	0.0018	8	15
	F	187.62	23.122	96.449	6.2021	16.712	0.7419	0.0088	0.2038	4.6040
	*p*	<0.00001	0.001341	<0.00001	0.037499	0.003495	0.414123	0.927568		
ABTS	SS	37.628	2.4090	36.594	0.0435	0.0276	0.2485	0.2211		
	Df	1	1	1	1	1	1	1	0.3248	77.499
	MS	37.628	2.4090	36.594	0.0435	0.0276	0.2485	0.2211	8	15
	F	926.79	59.335	901.33	1.0714	0.6798	6.1207	5.5458	0.0406	5.1666
	*p*	<0.00001	0.000057	<0.00001	0.330902	0.433549	0.038471	0.046322		

*x*_1_, *x*_2_ and *x*_3_, represent ethanol concentration, time and temperature. SOV-Source of variation; ^1^ Sum of squares; ^2^ Degree of freedom; ^3^ Mean of square; ^4^ *p* < 0.00001 highly significant; LP, lipid peroxidation inhibition; DPPH, DPPH radical scavenging activity; MC, metal chelating activity; OH, hydroxyl radical scavenging activity; ABTS, ABTS radical scavenging activity.

**Table 5:** Regression equations.

		R^2^	R_adj_^2^	CV (%)
LP	$y=24.181+2.516x_{1}-1.266x_{2}-2.519x_{3}-0.176x_{1}x_{2}-0.351x_{1}x_{2}x_{3}$	0.9974	0.9953	0.79
DPPH	$y=22.147+3.412x_{1}-1.477x_{2}-2.052x_{3}+0.152x_{1}x_{2}$	0.9981	0.9965	0.83
MC	$y=12.021+3.786x_{1}-1.561x_{2}-2.146x_{3}-0.504x_{1}x_{2}$	0.9921	0.9874	3.19
OH	$y=16.142+2.186x_{1}-0.767x_{2}-1.567x_{3}-0.397x_{1}x_{2}+0.652x_{1}x_{3}$	0.9764	0.9557	2.80
ABTS	$y=14.794+2.169x_{1}-0.549x_{2}-2.139x_{3}-0.176x_{2}x_{3}-0.166x_{1}x_{2}x_{3}$	0.9958	0.9921	1.36

**Table 6:** Values of kinetic parameters for antioxidant extraction.

			30%					50%	
	°C	b	k (1/min)	RMS (%)	R^2^	b	k (1/min)	RMS (%)	R^2^
LP	30	0.480	3.00 × 10^−3^	0.76	0.9948	0.384	2.24 × 10^−3^	1.21	0.9791
	40	0.500	3.31 × 10^−3^	4.34	0.8838	0.404	2.51 × 10^−3^	0.79	0.9906
	50	0.516	3.60 × 10^−3^	3.49	0.9099	0.422	2.82 × 10^−3^	1.18	0.9845
DPPH	30	0.254	3.63 × 10^−3^	3.26	0.9378	0.130	2.13 × 10^−3^	2.25	0.9274
	40	0.269	3.91 × 10^−3^	4.19	0.9258	0.141	2.53 × 10^−3^	1.50	0.9732
	50	0.280	4.10 × 10^−3^	4.57	0.9208	0.153	2.91 × 10^−3^	2.74	0.9201
MC	30	0.540	7.15 × 10^−3^	5.53	0.9485	0.332	4.78 × 10^−3^	4.55	0.9107
	40	0.559	7.55 × 10^−3^	5.13	0.9779	0.344	5.01 × 10^−3^	4.57	0.9281
	50	0.582	8.04 × 10^−3^	4.74	0.9839	0.362	5.31 × 10^−3^	3.86	0.9594
OH	30	0.360	5.26 × 10^−3^	5.33	0.9403	0.290	2.49 × 10^−3^	4.00	0.9076
	40	0.372	5.53 × 10^−3^	4.79	0.9573	0.315	2.80 × 10^−3^	3.98	0.8688
	50	0.390	5.75 × 10^−3^	5.67	0.9185	0.330	3.12 × 10^−3^	3.59	0.8739
ABTS	30	0.400	5.59 × 10^−3^	4.43	0.9185	0.296	3.68 × 10^−3^	3.04	0.9549
	40	0.416	5.81 × 10^−3^	3.76	0.9567	0.310	3.87 × 10^−3^	3.23	0.9600
	50	0.436	5.93 × 10^−3^	4.67	0.9413	0.332	4.23 × 10^−3^	1.74	0.9914

b—washing coefficient; k—slow-extraction coefficient; RMS—root mean square; R^2^—coefficient of determination. LP, lipid peroxidation inhibition; DPPH, DPPH radical scavenging activity; MC, metal chelating activity; OH, hydroxyl radical scavenging activity; ABTS, ABTS radical scavenging activity.

**Table 7:** Values of thermodynamic parameters for antioxidants extraction.

	30%					50%					
		E_a_^1^ (kJ/mol)	E_a_^2^ (kJ/mol)	ΔH* (kJ/mol)	ΔS* (K/Jmol)	ΔG* (kJ/mol)	E_a_^1^ (kJ/mol)	E_a_^2^ (kJ/mol)	ΔH* (kJ/mol)	ΔS* (K/Jmol)	ΔG* (kJ/mol)
LP	30	2.90	7.51	4.99	−277.79	89.17	3.85	9.36	6.85	−272.96	89.55
	40			4.91	−277.06	91.63			6.76	−273.41	92.34
	50			4.83	−277.34	94.41			6.68	−273.45	95.00
DPPH	30	3.98	4.97	2.45	−283.46	88.34	6.55	9.58	7.07	−272.65	89.68
	40			2.37	−283.77	91.19			6.98	−272.64	92.32
	50			2.28	−284.13	94.06			6.901	−270.23	94.18
MC	30	2.29	3.22	0.70	−283.59	86.63	3.82	4.26	1.74	−283.51	87.64
	40			0.62	−283.88	89.47			1.66	−283.49	90.39
	50			0.54	−283.94	92.25			1.57	−284.17	93.36
OH	30	3.26	3.63	1.11	−284.79	87.40	5.27	9.19	6.67	−272.65	89.29
	40			1.03	−285.16	90.28			6.59	−273.04	92.05
	50			0.95	−285.45	93.15			6.51	−273.32	94.79
ABTS	30	3.50	4.39	1.87	−281.78	87.25	4.57	5.90	3.15	−281.04	88.30
	40			1.79	−282.32	90.15			3.06	−281.62	91.21
	50			1.70	−282.85	93.06			2.98	−281.71	93.97

E_a_^1^ and E_a_^2^ represent activation energies for different extraction conditions. ΔH*, ΔS*, and ΔG* correspond to enthalpy, entropy, and Gibbs free energy changes, respectively. LP, DPPH, MC, OH, and ABTS are defined as in Table 3.

**Table 8:** Cytotoxic response to *E. carnea* L. extracts.

Ethanol %	T (°C)	Hep2c Cells ^a^ IC_50_ (μg/mL)	RD Cells ^b^ IC_50_ (μg/mL)	L2OB Cells ^c^ IC_50_ (μg/mL)
30	30	15.89 ± 0.94 ^d^	13.54 ± 0.35	24.59 ± 0.23
	40	14.34 ± 0.33	12.77 ± 0.41	23.34 ± 0.46
	50	13.34 ± 0.35	11.54 ± 0.48	22.23 ± 0.87
50	30	23.74 ± 0.76	19.88 ± 0.34	25.62 ± 0.87
	40	21.65 ± 0.44	18.55 ± 0.83	24.53 ± 0.44
	50	20.55 ± 0.23	17.49 ± 0.23	23.32 ± 0.74
cis-DDP ^e^		0.94 ± 0.55	1.4 ± 0.97	0.72 ± 0.64

^a^ Cell line derived from human cervix carcinoma. ^b^ Cell line derived from human rhabdomyosarcoma. ^c^ Cell line derived from murine fibroblast. ^d^ Mean value ± 3SD. ^e^ Cis-diamminedichloroplatinum.

**Table 9:** Antimicrobial activities (μg/mL) of *Erica carnea* L. extracts.

Stain	MIC	μg/L							
	30% Ethanol				50% Ethanol			A ^a^	N ^b^
	30 °C		40 °C	50 °C	30 °C	40 °C	50 °C		
*Staphylococcus aureus* ATCC 25923	19.53		19.53	19.53	156.25	78.125	39.1	39.1	
*Klebsiella pneumoniae* ATCC 13883	19.53		19.53	19.53	156.25	78.125	39.1	19.53	
*Escherichia coli* ATCC 25922	19.53		19.53	19.53	19.53	39.1	19.53	39.1	
*Proteus vulgaris* ATCC 13315	19.53		19.53	19.53	78.125	19.53	39.1	19.53	
*Proteus mirabilis* ATCC 14153	19.53		19.53	19.53	19.53	39.1	19.53	39.1	
*Bacillus subtilis* ATCC 6633	19.53		19.53	19.53	78.125	39.1	39.1	39.1	
*Candida albicans* ATCC 10231	19.53		19.53	19.53	156.25	19.53	78.125		19.53
*Aspergillus niger* ATCC 16404	19.53		19.53	19.53	39.1	19.53	19.53		39.1

^a^ A—Amracin (tetracycline hydrochloride); ^b^ N—Nystatin.

## Data Availability

Data are available from the corresponding author on request.
